# Young People’s Autonomy and Psychological Well-Being in the Transition to Adulthood: A Pathway Analysis

**DOI:** 10.3389/fpsyg.2020.01946

**Published:** 2020-08-07

**Authors:** Miguel Melendro, Gema Campos, Ana Eva Rodríguez-Bravo, Delia Arroyo Resino

**Affiliations:** ^1^Faculty of Education, Universidad Nacional de Educación a Distancia, Madrid, Spain; ^2^Faculty of Education, Universidad de Alcalá, Alcalá de Henares, Spain; ^3^Faculty of Education, Universidad Internacional de La Rioja, Logroño, Spain

**Keywords:** young people, well-being, autonomy, education, employment, disadvantage

## Abstract

Young people transition to adulthood via diverse pathways; among the most significant are those dominated by education, employment, or social disadvantage. These pathways are determined, to a large extent, by the level of well-being and autonomy young people develop to help them face their own realities. The aim of this study is to analyze the relationship between young people’s psychological well-being and autonomy – key factors in the transition to adulthood – and the relationship these factors have with the main pathways followed during transition. To this end, Ryff’s Model of Psychological Well-being and the Transition to Adulthood Autonomy Scale (EDATVA) were used to evaluate a total of 1148 Spanish and Colombian subjects aged between 16 and 21. Correlations and differences between scores were subsequently analyzed. Subjects were also asked to identify the most relevant aspect of their transition to adulthood as either education, employment, or social disadvantage. Results from all three pathways for transition to adulthood show a trend in which higher levels of well-being correspond to higher levels of autonomy. In general, the results for the young people on the education pathway show high levels of autonomy and well-being, as well as a significantly higher level of critical thinking compared to young people on other pathways. The scores from employed young people reveal a greater capacity for self-organization in relation to the other two pathways. The results for disadvantaged young people show significantly greater socio-political engagement than that of young people on the education and employment pathways. However, the disadvantaged group also displays the lowest level of psychological well-being. These results provide elements for a better understanding of young people’s different transition pathways to adulthood and constitute an important point of reference for future research. They also provide data that may be relevant in guiding potential educational, psychological, and social interventions within this population group.

## Introduction

The period between the ages of 16 and 21 spans the end of adolescence and the onset of a developmental stage that was recently termed *emerging adulthood* (Arnett, 2007; Berger, 2016; Maree and Twigge, 2016). Throughout this stage, social relationships, especially family and peer relationships, play an important role in how young adults develop well-being and autonomy (Arnett, 2015; Campione-Barr et al., 2015; Davies et al., 2015; Jorgensen and Nelson, 2018). Some researchers examining family settings have underlined the overarching and complex relationship between parental control, the family’s approach to supporting autonomy, and the different pathways these *emerging adults* will follow (Liga et al., 2018). Peer relationships in early adolescence also provide opportunities for developing one’s autonomy (Oudekerk et al., 2015). This developmental stage is also understood as a period of instability in which an adolescent becomes an autonomous adult by facing unfamiliar situations, mainly in academic settings and the workplace (Arnett, 2015).

Three social institutions – family, school, and the job market – will determine the main pathways by which young people transition to adulthood, whether by entering the workforce or by prolonging education to join the workforce at a later time (Settersten et al., 2015; Arnett and Tanner, 2016; Schoon and Lyons-Amos, 2016). However, structural dysfunction that prevents families from fulfilling their protective and educational roles gives rise to a third pathway characterized by social disadvantage. While less socially visible than the others, this pathway is no less significant. As stated by Munson et al. (2013), socially disadvantaged youth must be studied if we are to understand how they experience the processes of emerging adulthood; until now, such studies have been limited almost exclusively to students and workers.

### Psychological Well-Being and Autonomy in the Transition to Adult Life

The term *well-being*, referring to an optimal psychological state, is a construct that may be defined from two complementary perspectives. The hedonic perspective frames well-being as the positive assessment of one’s own life, and it has been linked to a sense of satisfaction and emotional stability, as well as to subjective well-being. Eudaimonic well-being, which corresponds to psychological well-being, emphasizes personal development, self-fulfillment, exploring one’s own potential and planning for the future, and contributing to the well-being of others (Deci and Ryan, 2008; Ryff and Singer, 2008; Waterman et al., 2010; Adler and Seligman, 2016). Recent studies have underlined the differences between these two perspectives by examining their links to different character strengths, such as hope, zest, gratitude, curiosity, and love (Hausler et al., 2017). They have also found temporal differences between these perspectives; over the long-term, psychological well-being, rather than subjective, is a more robust predictor of future well-being (Joshanloo, 2019). This concept of eudaimonic or psychological well-being is the one we employ in our study. Here, psychological well-being is examined using the Ryff Scale of Measurement, which is based on Ryff’s six-factor model (1989) with its dimensions of self-acceptance, positive relations, autonomy, environmental mastery, personal growth, and purpose in life.

More recently, Ryff (2018) provided a theoretical exploration of autonomy from the standpoint of self-determination theory and linked to eudaimonic well-being; she also stressed the need for a tool able to assess autonomy according to this expanded view. We agree with the conceptualization of autonomy as a complex process subject to continuous remodeling over a lifetime and through interactions with others (Muñoz-López and Alvarado, 2011; Cáliz et al., 2013; Posada, 2013; Inguglia et al., 2014; Bernal Romero et al., 2020b). Having established this basis, a thorough review of the literature and measurement tools addressing autonomy provides a definition according to three processes: one that is personal, subjective, or intrasubjective; an intersubjective process, involving another person; and, lastly, a trans-subjective process referring to interactions between the subject and the surrounding community or society (Bernal Romero et al., 2020b). This definition deviates from the classic notion of autonomy as self-sufficiency or the capacity to manage exclusively on one’s own (Delbosc and Vella-Brodrick, 2015; Garberoglio et al., 2017). Rather, it focuses on interactions with other individuals, and with society as a whole by including community and socio-political engagement (Parron, 2014). This perspective raises new challenges for the concept of emerging adulthood, which focuses on personal or intrasubjective processes including assuming responsibility for oneself, making decisions independently, and achieving financial independence (Settersten et al., 2015).

The relationship between well-being and autonomy has been studied from many distinct perspectives (Schüler et al., 2014; Legault et al., 2017; Ryff, 2018). Multiple authors have confirmed a link between these two variables and determined that promoting autonomy may increase the level of well-being (Reis et al., 2000; Inguglia et al., 2014; Delbosc and Vella-Brodrick, 2015; Alivernini et al., 2019; Chatzisarantis et al., 2019). However, it has also been shown that young people score below other age groups on the *autonomy* dimension, as well as on the dimensions *self-acceptance* and *environmental mastery* (Meléndez et al., 2018). To better understand these results, we should be mindful that *self-acceptance* is a trait associated with mature personalities (Allport, 1961); *autonomy* and *environmental mastery* are other dimensions for which scores tend to increase with age (Ryff, 1989, 1991; Ryff and Keyes, 1995). Generally speaking, young people score higher than do other age groups for *positive relations* since they are less inhibited about making social contact (Mayordomo et al., 2016; Meléndez et al., 2018). The same effect can be seen in the score for *personal growth*, which is similar between young people and adults but much higher in both groups than in the elderly; this finding is linked to having a longer time span in which to discover and pursue personal interests and goals (Mayordomo et al., 2016).

### Pathways in Emerging Adulthood

Three social institutions exert an influence on the structural setting in which adolescent development takes place: the family, the school, and the job market (INJUVE, 2017). This observation also appears in national and international statistics designed to measure young people’s living conditions (UNESCO, Eurostat, Eurofound, Eurydice, CEPAL, INJUVE). Schoon and Lyons-Amos (2016) classify the pathways pertaining to this stage of development according to the age at which emerging adults leave formal education and begin work, and by their level of job security. Young people’s family environments play a major role in their choice of pathway. With this reference framework in mind, it is possible to state that emerging adults follow three transitional pathways: education, employment, and social disadvantage.

The education pathway describes the situation of young people who delay their transition to adult life and postpone entry into the workforce by extending their academic years. This transcultural phenomenon promotes the transformation of an industrial economy into an information-based economy (Rifkin, 2011; Arnett and Tanner, 2016; Wood et al., 2018). Recent studies show that the school-to-university transition process has a marked impact on the overall psychological well-being of the emerging adults on the educational pathway. Its effects are mainly seen on such components of well-being as personal growth, with another weaker link to personal autonomy (Barrantes-Brais and Ureña-Bonilla, 2015; Malinauskas and Dumciene, 2017).

In contrast, the young people transitioning to adulthood on the employment pathway are those who are actively working or else participating in short-term vocational training programs not offered by universities. We know that these emerging adults have less privileged backgrounds compared to those who prolong their studies (Schoon and Lyons-Amos, 2016). The employment pathway has become even more relevant in the new millennium in light of the unemployment that may create lifelong obstacles for this generation (European Policy Centre, 2014; Pimentel et al., 2016). Autonomy, meaning self-governance and responsible self-control, is the dimension with the strongest effect on psychological well-being in this group (Fotiadis et al., 2019). Furthermore, young people who have found jobs matching their skill sets score higher in the areas of resilience, optimism, autonomy, environmental mastery, self-efficacy, and overall life satisfaction, with fewer indicators of anxiety than are shown by their employment-pathway counterparts who are still seeking jobs (Merino et al., 2019). These findings emphasize the need to train these individuals in how to develop and strengthen the resources that will contribute to their psychological well-being, and which are largely linked to the development of emotional intelligence (Di Fabio and Kenny, 2016). Specific training in the acquisition of concrete job-finding skills may be especially valuable since it has a positive influence on young peoples’ understanding of how to handle themselves in the workplace (Guichard et al., 2012; Maree and Twigge, 2016; Zacher and Schmitt, 2016; Merino et al., 2019).

Lastly, the social disadvantage pathway is marked by the persistent influence of obstacles to social inclusion that severely hamper the individual’s emerging adulthood; such obstacles may include lack of family support, academic failure, and the inability to hold down a job (Munson et al., 2013; Settersten et al., 2015; Cameron et al., 2018; Cuenca et al., 2018; García-Castilla et al., 2018). Indicators consistently show that these young people have not benefitted from the same level of access to both educational and professional opportunities (Schoon and Lyons-Amos, 2016). Not all of these socially disadvantaged youths – specifically those receiving long-term social support – are able to live in a family setting. In Europe alone, more than a million minors live in residential facilities or with foster families (Cameron et al., 2018). Many of these adolescents transition prematurely to adulthood without benefitting from the trial-and-error period common to other adolescents who enjoy family support (López et al., 2013; Munson et al., 2013; Soldevila et al., 2013; Singer and Berzin, 2014; Settersten et al., 2015; Campos et al., 2020). In addition, they display very different ways of coping with social interactions, some of which involve professional care workers (Schofield et al., 2016). One trait shared by socially disadvantaged young people is their more external locus of control combined with a greater sense of lack of control over their own lives (Król et al., 2019).

## Materials and Methods

### Specific Objectives

As stated above, the pathway followed – education, employment, or social disadvantage – will largely be determined by the degree of autonomy and well-being present in a young person facing the future. With this in mind, the study aims are as follows: *to analyze, in emerging adults, the relationship between autonomy and psychological well-being* – *two key factors in the processes of transitioning to adulthood* – *and the way these factors differ according to the main pathways young people follow in their transition.*

Two specific objectives must also be met in order to complete the larger analysis: first, to examine the relationship between dimensions on the autonomy scale and dimensions on the well-being scale for each distinct pathway (education, employment, and social disadvantage), considered independently, and second, to detect any significant differences between emerging adults on different pathways using scores from each dimension on the autonomy questionnaire, and from each dimension on the well-being questionnaire.

### Participants

The sample was selected using a purposive (non-probability) sampling method and participation was voluntary. Inclusion criteria were identification with one of the three different pathways (education, employment, and social disadvantage), age between 16 and 21 years, association with an institution (academic, employment, or social), and the ability to read and write. Potential participants with a recognized functional, physical, or mental disability were excluded.

Pathways were identified as the type of transition to adult life, in accordance with the following definitions: (1) Education pathway: emerging adults dedicated to academic studies aimed at obtaining a university degree; (2) Employment pathway: emerging adults who are actively working or enrolled in short-term vocational training programs, outside the university framework; and (3) Social disadvantage: emerging adults benefitting from long-term formal social support services.

Each itinerary has elements that characterize it and that differentiate it from the others, without them being isolated categories; they are not exclusive. For example, the fact that a young person belongs to the itinerary of social difficulty does not mean that he is neither studying nor working, but rather that what characterizes him, for the definition of this sample, are the difficulties of social origin in his/her development. In the same way, a young person who is working, inserted in a job, may have notable family problems, but what characterizes him or her, for the purpose of our sample, is that he or she is currently working or in a professional training institution.

The final sample consisted of 1148 emerging adults from Spain (Madrid) and Colombia (Bogota); although the sample included young people from two different countries, we did not detect dependence between the country and each of the pathways (chi = 14.596; *p* = 0.132). Participants’ ages ranged from 16 to 21 years (mean = 18.20; *SD* = 1.8) and about 60% were female. The education pathway accounted for 66.2% of the participants (48.6%, last years in high schools; 51.4% universities). The employment pathway accounted for 21.5% (42.1% in first- and second-cycle vocational training and 57.9% in active work). The remaining 12.3% were in the social disadvantage pathway (41.1% attended day centers and occupational and labor integration workshops, and 58.9% attended residential institutions for minors protected by the State). Table 1 below lists the sample characteristics for each pathway.

**TABLE 1:** Sample characteristics.

Variables/Pathways		Education	Employment	Social disadvantage
Sex	Male	311 (40.9%)	74 (30%)	69 (48.9%)
	Female	448 (58.9%)	173 (70%)	71 (50.4%)
Country	Spain	317 (41.7%)	107 (43.3%)	57 (40.4%)
	Colombia	443 (58.3%)	140 (56.7%)	84 (59.65)
Age		*M* = 18.11 (*SD* = 1.8)	*M* = 18.5 (*SD* = 1.42)	*M* = 18.16 (*SD* = 1.28)
Total *N*		760	247	141

### Tools Used

#### Assessment of Autonomy in Emerging Adults

Autonomy in our population of emerging adults was measured using the Autonomy Scale for Transition to Adult Life (Bernal Romero et al., 2020a). The scale comprises 19 items grouped into four dimensions, which are described below; the Cronbach’s alpha coefficient is given for each dimension in this study. *Self-organization ability* (six items, α = 0.80): This dimension of autonomy is self-focused since it is present in subjects able to carry out metacognitive tasks, i.e., thinking about what they are learning, organizing effectively, and planning future learning processes. *Ability to analyze context* (four items, α = 0.71): Here, autonomy remains focused on the self, but within the framework of wider systems, such as social and political settings. An individual able to analyze context will search for information, mull over proposals, and develop opinions regarding personal, social, and political decisions. *Critical thinking* (five items, α = 0.70): This dimension of autonomy also focuses on the self and revolves around one’s own ideas and socially recognized rights. Critical thinking skills are related to respect for the self and for one’s opinions and ideas and include the ability to express these ideas during a confrontation or a situation requiring an assertive response. *Socio-political engagement* (four items, α = 0.77) is a community-focused dimension that assumes that exercising autonomy may affect external systems, and not only the subject (Bernal Romero et al., 2020a). Cronbach’s alpha for this set of items was 0.84 for the full sample. All dimensions were measured on a four-point Likert scale with 1 indicating total disagreement and 4 indicating total agreement. Factor analysis confirmed a structure based on four factors (*self-organization, context analysis, critical thinking*, and *socio-political engagement*) including a total of 19 items with goodness-of-fit indexes of 0.93 by CFI and 0.90 by NNFI; the RMSEA was 0.05 and the SRMR was 0.06. Given the above, we can state that the model fit the data well.

#### Assessment of Well-Being in Emerging Adults

Well-being was measured using the Spanish-language adapted version of the Ryff Psychological Well-being Scale published by Díaz et al. (2006). The scale comprises 39 items grouped into six dimensions, which are listed below along with their Cronbach’s alpha coefficients. *Self-acceptance* (six items, α = 0.81) is a key component of well-being. This trait enables one to feel comfortable with oneself despite being fully aware of shortcomings. *Positive relations* (six items, α = 0.76) refers to possessing quality relationships with others. *Autonomy* (eight items, α = 0.71) indicates a person’s ability to maintain his/her individuality within different social contexts. *Environmental mastery* (six items, α = 0.63) is associated with the internal locus of control and a high degree of self-efficacy. This trait is associated with social coherence – a perception of the world as discernible, predictable, and logical and therefore controllable – and with concern for and interest in the larger community. *Personal growth* (seven items, α = 0.62) refers to a person’s positive learning and development. *Purpose in life* (six items, α = 0.81) means having goals and the impression of moving forward in life, associated with motivation to act and evolve. The total reliability of the scale is 0.91. The well-being scale includes a total of 17 reverse-score items (2, 4, 5, 8, 9, 13, 15, 20, 22, 25, 26, 27, 29, 30, 33, 34, and 36). Participants submitted their responses using Likert scale scores ranging from 1 (totally disagree) to 6 (totally agree) (Díaz et al., 2006, p. 573). Confirmatory factor analysis revealed a good fit to a structure with six factors (*self-acceptance*, *positive relations*, *autonomy*, *environmental mastery*, *personal growth*, and *purpose in life*). Goodness-of-fit indices were acceptable, with values of 0.91 by CFI and 0.92 by NNFI; the RMSEA value was 0.04 and the SRMR value was 0.05. These results suggest that the proposed model fits the data well.

#### Procedure and Data Analysis

Questionnaires were completed between late 2019 and early 2019, either on paper or in digital format. Participants filled out questionnaires during group sessions that were held during class time (when given at academic institutions) or during break times (at workplaces and social resource centers). Each questionnaire took approximately 45 min to complete. Participation was voluntary and each participant read and signed an informed consent form. No remuneration was offered or given. Participants, and the legal guardians of participating minors where applicable, were informed regarding the purpose of the study and given detailed instructions. Once the study had been approved by human research ethics committees at the participating universities, researchers adhered to the protocol established by the Declaration of Helsinki (64th general assembly of the WMA, Brazil, October 2013). Answer confidentiality was guaranteed to eliminate experimenter bias.

Once data had been gathered and the databases prepared, we focused on the first specific objective of the study: examining the relationships between dimensions on two scales, one measuring autonomy (*self-organization, personal growth, context analysis, critical thinking*, and *socio-political involvement*) and the other measuring well-being (*self-acceptance, positive relations, autonomy, environmental mastery, personal growth*, and *purpose in life*). The study population was divided into three comparison groups or pathways (education, employment, and social disadvantage) and we determined the Pearson correlation for each pathway. The strength of correlation values was interpreted as follows: 0.20 and lower, very low; 0.21–0.40, low; 0.41–0.70, moderate; 0.71–0.90, high; 0.91–1, high (Mateo, 2004).

Next, to fulfill the second specific purpose of this study, we performed inferential tests to detect any significant differences associated with the pathways followed by emerging adults (education, employment, or social disadvantage), using their scores on each dimension of the autonomy scale and on each dimension of the well-being scale. Before carrying out these tests, we checked for normal distribution and homogeneity of variance (homoscedasticity) between populations as recommended by Pardo and San Martin (2010). Since results from the autonomy scale did not meet the assumptions of normality or homoscedasticity, we checked for differences between the three pathways using the Kruskal–Wallis test, followed by the Bonferroni-corrected Mann–Whitney *U*-test. The Bonferroni correction, used when more than two comparison groups are present, indicates a statistically significant difference between two groups when the critical value (*p*-value) is less than an alpha of 0.01 (Pardo and San Martin, 2010). Results from this test are displayed as means, in addition to average ranges, to facilitate interpretation. Since results on the well-being scale met the assumptions of normality and homoscedasticity, they could be compared using one-way ANOVA, a parametric test. After obtaining these results, we determined the magnitude of the differences by calculating the effect size using Cohen’s *d* method, according to which values near 0.20 indicate a small effect size, values near 0.50 indicate a medium effect size, and values near 0.80 indicate a large effect size (Cohen, 1992). Analyses were performed using SPSS version 25.0 (IBM SPSS Statistics 25), with G^∗^Power 3.1 to calculate effect size.

## Results

The results of specific objectives 1 and 2 are presented below. As mentioned above, although the sample included young people from two different countries, we did not detect dependence between the country and each of the pathways. Likewise, no significant differences were found in the well-being or autonomy scores, in the different pathways, due to sex or age, so these variables were not controlled.

### Specific Objective 1: Relationship Between Dimensions on the Autonomy and Well-Being Scales by Pathway

#### Education Pathway

Table 2 addresses our first specific objective by showing relationships between dimensions on the autonomy and well-being scales for the education pathway. First of all, we see that the *self-organization* dimension on the autonomy scale is closely correlated to two dimensions on the well-being scale: *environmental mastery* and *purpose in life* (*r* = 0.463 and *r* = 0.577, respectively). Both correlations are positive and significant (*p* < 0.01), and the same relationship is present between *self-organization* and *purpose in life*. The correlation strengths in both cases are moderate according to Mateo (2004). Additional correlations of interest, although with lower correlation strengths, can be found between *self-organization* and the well-being scale dimensions *self-acceptance* (*r* = 0.377), *autonomy* (*r* = 0.210), and *personal growth* (*r* = 0.382).

**TABLE 2:** Pearson correlations between autonomy scale and well-being scale dimensions, all pathways.

	Self-acceptance	Positive relations	Autonomy	Environmental mastery	Personal growth	Purpose in life	Total well-being
**Education pathway**							
Self-organization	0.377**	0.069	0.210**	0.463**	0.382**	0.577**	0.451**
Context analysis	0.274**	0.206**	0.404**	0.330**	0.331**	0.323**	0.416**
Critical thinking	0.175**	0.066	0.222**	0.242**	0.278**	0.289**	0.282**
Socio-political engagement	0.170**	0.130**	0.108**	0.194**	0.115**	0.188**	0.203**
Total autonomy	0.340**	0.167**	0.319**	0.418**	0.372**	0.464**	0.459**
**Employment pathway**							
Self-organization	0.408**	0.081	0.191**	0.516**	0.264**	0.577**	0.460**
Context analysis	0.199**	0.208**	0.355**	0.293**	0.319**	0.318**	0.400**
Critical thinking	0.046	–0.045	0.195**	0.082	0.192**	0.134*	0.155*
Socio-political engagement	0.086	0.013	–0.004	0.134*	0.076	0.158*	0.107
Total autonomy	0.253**	0.083	0.252**	0.354**	0.301**	0.413**	0.387**
**Disadvantage pathway**							
Self-organization	0.290**	0.000	0.136	0.288**	0.349**	0.527**	0.350**
Context analysis	0.355**	0.226**	0.289**	0.344**	0.470**	0.402**	0.471**
Critical thinking	0.123	0.094	0.062	0.385**	0.302**	0.344**	0.277**
Socio-political engagement	0.224**	0.080	–0.129	0.172*	0.139	0.317**	0.160
Total autonomy	0.322**	0.133	0.106	0.398**	0.411**	0.524**	0.410**

*p < 0.05; **p < 0.01.

The strongest correlation for the *context analysis* dimension is with the *autonomy* dimension (*r* = 0.404). This correlation is statistically significant (*p* < 0.01). Other significant correlations for *context analysis* were with *positive relations* (*r* = 0.206), *environmental mastery* (*r* = 0.330), *personal growth* (*r* = 0.331), and *purpose in life* (*r* = 0.323).

The *critical thinking* dimension displayed its strongest correlations with two dimensions on the well-being scale: *personal growth* and *purpose in life* (*r* = 0.278 and *r* = 0.289). Both correlations were significant (*p* < 0.01), but the correlation strength was low (Mateo, 2004). Correlations between this dimension and *autonomy* (*r* = 0.222), *environmental mastery* (*r* = 0.248), and *self-acceptance* (*r* = 0.175) were also significant (*p* < 0.01), although correlation strength was somewhat lower.

The strongest correlations for *socio-political engagement* were with *environmental mastery* (*r* = 0.194; *p* < 0.01) and *purpose in life* (*r* = 0.108; *p* < 0.01), although correlations between *socio-political engagement* and all dimensions on the well-being scale were significant.

Lastly, we must underline that each of the dimensions on the autonomy scale was significantly and positively correlated with the total score on the well-being scale. The dimensions with the closest correlations to that total were *self-organization* (*r* = 0.451; *p* < 0.01) and *context analysis* (*r* = 0.416; *p* < 0.01). We also observe a positive and significant correlation between the total autonomy and the total well-being scale scores (*r* = 0.459; *p* < 0.01).

#### Employment Pathway

The middle section of Table 2 displays the relationships between dimensions on the autonomy and the well-being scales for the employment pathway.

In this group, the *self-organization* dimension on the autonomy scale correlates the closest to two dimensions on the well-being scale: *environmental mastery* and *purpose in life* (*r* = 0.516 and *r* = 0.577, respectively). Both correlations are positive and significant (*p* < 0.01) with a moderate correlation strength (Mateo, 2004). There was another close correlation between *self-organization* and *self-acceptance* (*r* = 0.408).

The *context analysis* dimension was most closely correlated with the *autonomy* dimension (*r* = 0.355; *p* < 0.01), followed by *personal growth* (*r* = 0.319; *p* < 0.01) and *purpose in life* (*r* = 0.318; *p* < 0.01). Furthermore, the correlations between *context analysis* and all dimensions on the well-being scale were significant.

The closest correlation for the *critical thinking* dimension was with the *autonomy* dimension on the well-being scale (*r* = 0.195; *p* < 0.01), followed by a similar correlation to the *personal growth* dimension (*r* = 0.192; *p* < 0.01).

The *socio-political engagement* dimension was most closely correlated with *purpose in life* (*r* = 0.158; *p* < 0.05), although the correlation strength was low (Mateo, 2004); the only other dimension of well-being to correlate significantly with *socio-political engagement* was *environmental mastery* (*r* = 0.134; *p* < 0.05).

Lastly, we underline that each of the dimensions on the autonomy scale, except for *socio-political involvement* (*p* < 0.05), shows a significant and positive correlation with the total score on the well-being scale. The greatest correlation strengths for both this pathway and the education pathway were between the total well-being score and the *self-organization* dimension (*r* = 0.460; *p* < 0.01), and between total well-being and the *context analysis* dimension (*r* = 0.400; *p* < 0.01) on the autonomy scale. Furthermore, there is a significant and positive relationship between total score on the autonomy scale and total score on the well-being scale (*r* = 0.387; *p* < 0.01).

#### Social Disadvantage Pathway

To conclude with our first specific objective, the third section of Table 2 presents the relationship between dimensions on the two scales for the social disadvantage pathway.

These results show that the *self-organization* dimension on the autonomy scale correlates the closest with the *purpose in life* dimension on the well-being scale (*r* = 0.527; *p* < 0.01). The correlation is moderate (Mateo, 2004), and there are other moderately strong significant correlations between *self-organization* and *self-acceptance* (*r* = 0.290; *p* < 0.01), *environmental mastery* (*r* = 0.288; *p* < 0.01), and personal growth (*r* = 0.349; *p* < 0.01).

Although correlations between *context analysis* and every dimension on the well-being scale were all significant (*p* < 0.01), the strongest correlations were with *personal growth* (*r* = 0.470; *p* < 0.01) and *purpose in life* (*r* = 0.402; *p* < 0.01). Correlation strengths in both cases were moderate (Mateo, 2004).

The closest correlations for the *critical thinking* dimension were with the *environmental mastery* and *purpose in life* dimensions (*r* = 0.385 and *r* = 0.344, respectively); both correlations were significant (*p* < 0.01) and positive. A similar correlation was also present for the personal growth dimension (*r* = 0.302; *p* < 0.01).

The *socio-political engagement* dimension correlated the closest to the *purpose in life* dimension (*r* = 0.317; *p* < 0.01); the correlation with *environmental mastery* (*r* = 0.172; *p* < 0.05) was also significant.

Our last remark on the social disadvantage pathway is that all dimensions on the autonomy scale, except for *socio-political engagement*, displayed significant and positive correlations with the total score on the well-being scale. These observations were also true for the employment pathway. The highest correlation strengths for this pathway, as for the education and employment pathways, were between the total autonomy scale score and the *self-organization* dimension (*r* = 0.350; *p* < 0.01) and between the total autonomy score and the *context analysis* dimension (*r* = 0.471; *p* < 0.01) on the well-being scale. Likewise, there is a significant and positive relationship between the total scores on the autonomy and the well-being scales (*r* = 0.410; *p* < 0.01), with a moderate correlation strength (Mateo, 2004).

### Specific Objective 2: Score Comparisons Across Pathways

#### Autonomy Scale

Since results from the autonomy scale did not meet the assumption of normality, we used non-parametric tests (Kruskal–Wallis) to identify any differences between the education, employment, and social disadvantage groups on each of the different dimensions of the autonomy scale.

Table 3 displays the *H* statistic of the Kruskal–Wallis test (chi-square) for each of the dimensions on the autonomy scale and for the total score. This statistic is associated with a critical value, or *p-*value, which is less than 0.05 for each dimension on the autonomy scale. This value lets us reject the null hypothesis and conclude that scores obtained on these dimensions vary among the different pathways being compared. Nevertheless, there are no differences between groups for the total scores on each scale.

**TABLE 3:** Descriptive statistics and Kruskal–Wallis test.

		*N*	Self-organization	Context analysis	Critical thinking	Socio-political engagement	Total autonomy
Mean rank	Education	760	552.56	567.44	609.00	575.98	582.53
	Employment	247	635.39	618.31	549.85	526.58	577.16
	Disadvantage	141	586.06	535.79	431.70	650.47	526.55
Chi-squared			11,930	6718	36,100	12,709	3,414
Df			2	2	2	2	2
Asympt. sig. (bivariate)			0.003	0.035	0.000	0.002	0.181

We applied the Bonferroni-corrected Mann–Whitney *U*-test to determine which groups showed differences in scores on each scale dimension.

First, we compared scores on each dimension of the autonomy scale between the education and employment groups. Table 4 shows that the only dimensions with statistically significant differences between these two pathways, referring to *p*-values of less than 0.01, are *self-organization* and *critical thinking*. The employment group $\left(\bar{\text{X}}=20.44;SD=3.40\right)$ scores higher than the education group for self-organization, whereas the education group $\left(\bar{\text{X}}=19.65;SD=3.36\right)$ scores higher for critical thinking $(\bar{\text{X}}$ = 19.79; *SD* = 3.64). No other dimensions revealed significant differences between the education and employment groups. According to Cohen’s classification (1992), effect size (*d*) is small for both dimensions.

**TABLE 4:** Descriptive statistics and Mann–Whitney *U-*test for each pathway.

		*N*	Self-organization	Context analysis	Critical thinking	Socio-political engagement
Mean rank	Education	760	486.01 ($\bar{\text{X}}$ = 19.65; *SD* = 3.36);	492.94 ($\bar{\text{X}}$ = 20.25; *SD* = 3.51)	516.91 ($\bar{\text{X}}$ = 19.79; *SD* = 3.64)	514.66 ($\bar{\text{X}}$ = 13.59; *SD* = 4.66)
	Employment	247	559.36 ($\bar{\text{X}}$ = 20.44; *SD* = 3.40)	538.04 ($\bar{\text{X}}$ = 20.75; *SD* = 3.37)	464.27 ($\bar{\text{X}}$ = 19.04; *SD* = 3.99)	471.21 ($\bar{\text{X}}$ = 12.90; *SD* = 4.44)
Mann–Whitney *U-*test			80,185.500***	85,452.000	84,046.000**	85,762.000**
Effect size (*d*)			0.218		0.156	
Mean rank	Education	760	447.06	455.01	472.59	441.83
			472.26 ($\bar{\text{X}}$ = 19.73;	429.41 ($\bar{\text{X}}$ = 19.73;	334.63 ($\bar{\text{X}}$ = 17.04;	500.45 ($\bar{\text{X}}$ = 14.68;
	Disadvantage	141	*SD* = 4.23)	*SD* = 4.21)	*SD* = 4.24)	*SD* = 5.19)
Mann–Whitney *U* test			50,583.000	50,536.000	37,171.500***	46,607.000**
Effect size (*d*)					0.393	0.164
Mean rank	Employment	247	200.03	204.27	209.59	179.36
	Disadvantage	141	184.81	177.38	168.07	221.01
Mann–Whitney			16,047.000	14,999.500	13,687.500***	13,675.000***
*U*-test Effect size (*d*)					0.362	0.363

**p < 0.01; ***p < 0.001.

Table 4 also lists results from comparisons between the education and social disadvantage groups. Here, differences between the two groups are present for the *critical thinking* and *socio-political engagement* dimensions (*p* < 0.01). Higher scores for *critical thinking* pertain to the education group ($\bar{\text{X}}=19.79;\,S\,D=3.64$), whereas higher scores for *socio-political engagement* belong to the social disadvantage group ($\bar{\text{X}}=14.68;\,S\,D=5.19$). Effect size is between low and moderate for the *critical thinking* dimension, and low for the *socio-political engagement* dimension.

The final comparison examines autonomy scale scores between the employment and social disadvantage groups. At the bottom of Table 4, we find differences between these two groups (*ð* < 0.01) for the *critical thinking* dimension: the employment group ($\bar{\text{X}}=19.04;\,S\,D=3.99$) scored higher than the social disadvantage group ($\bar{\text{X}}=17.04;\,S\,D=4.24$), while the social disadvantage group ($\bar{\text{X}}=14.68;\,S\,D=5.19$) has higher scores for *socio-political engagement*.

#### Well-Being Scale

Since results from the well-being scale met the assumption of normality, we used a one-way ANOVA test to compare scores for each of the dimensions on that scale between different pathways.

Table 5 displays a summary of ANOVA results in which the value of the *F* statistic is associated with the critical value or *p*-value. Since this value remains lower than 0.05 for all dimensions and for the total score, it is reasonable to reject the equality of means hypothesis and therefore conclude that scores for each dimension on the well-being scale, as well as total scores, are not equivalent among the three population groups.

**TABLE 5:** Summary of one-way ANOVA with multiple *post hoc* comparisons: Scheffé’s method.

Dependent variable	*F*	(I) Pathway	(J) Pathway	Difference in means (I-J)	Standard error	Sig.	*d*
Self-acceptance	14.712	Education	Employment	0.10988	0.417	0.966	
			Disadvantage	2.832*	0.527	0.000	0.532
		Employment	Disadvantage	2.722*	0.606	0.000	0.491
Positive relations	5.589	Education	Employment	−0.520	0.450	0.513	
			Disadvantage	1.626*	0.569	0.017	0.445
		Employment	Disadvantage	2.146^∗^	0.654	0.005	0.364
Autonomy	8.880	Education	Employment	−0.995	0.499	0.137	
			Disadvantage	2.057*	0.631	0.005	0.302
		Employment	Disadvantage	3.053*	0.725	0.000	0.429
Environmental mastery	15.811	Education	Employment	−0.742	0.373	0.139	
			Disadvantage	2.258*	0.472	0.000	0.444
		Employment	Disadvantage	3.001*	0.543	0.000	0.574
Personal growth	10.092	Education	Employment	−0.469	0.384	0.476	
			Disadvantage	1.954*	0.487	0.000	0.372
		Employment	Disadvantage	2.423*	0.560	0.000	0.456
Purpose in life	8.680	Education	Employment	−0.699	0.419	0.249	
			Disadvantage	1.815*	0.530	0.003	0.314
		Employment	Disadvantage	2.514*	0.609	0.000	0.445
Total well-being	17.917	Education	Employment	−3.268	1.894	0.226	
			Disadvantage	12.685*	2.397	0.000	0.487
		Employment	Disadvantage	15.953*	2.754	0.000	0.642

*p < 0.05.

We find significant differences between the education and social disadvantage groups (*p* < 0.05) for the *self-acceptance* dimension: the education group scores higher and the effect size is moderate (*d* = 0.532). The employment group also scores higher than the social disadvantage group with a Cohen’s *d* of 0.491. The education group scores higher than the social disadvantage group on the *positive relations* dimension, with an effect size of 0.445. The employment group also scores higher than the social disadvantage group, with *d* = 0.364. For the *autonomy* dimension, we observe higher scores in the education group than in the social disadvantage group, with an effect size of 0.302; the employment group also scores higher than the social disadvantage group (*d* = 0.429). The education group scored higher than the social disadvantage group on the *environmental mastery* dimension (*d* = 0.444). The employment group also scored higher than the social disadvantage group, with a moderate effect size (*d* = 0.574). Regarding significant differences for *personal growth*, the education group scored higher than the social disadvantage group (*d* = 0.372) and so did the employment group (*d* = 0.456). Scores for the last dimension on the well-being scale, *purpose in life*, differ significantly between the education and social disadvantage groups (higher in the education group, *d* = 0.314). The employment group scores higher than the social disadvantage group with an effect size between low and moderate (*d* = 0.445). Lastly, the education group scores higher than the social disadvantage group on the well-being scale total, with *d* = 0.487; the employment group scores higher than social disadvantage with *d* = 0.642, an effect size between moderate and high. We therefore see a repeating pattern: where significant differences exist between the social difficulty and education groups, the education group scores higher; where such differences exist between employment and social difficulty, the employment group scores higher.

## Discussion

### Relationship Between Dimensions on the Autonomy Scale and the Well-Being Scale by Pathway (Specific Objective 1)

Returning to the first specific objective of our study – *to examine the relationship between dimensions on the autonomy scale and dimensions on the well-being scale for each distinct pathway (education, employment, and social disadvantage), considered independently* – the results show positive and significant correlations between the dimensions on these two scales, for each of the three pathway groups.

One initial finding from this study is that for all three pathways, higher levels of well-being correspond to higher levels of autonomy and vice versa. This finding coincides with results from similar studies (Delbosc and Vella-Brodrick, 2015; Alivernini et al., 2019; Chatzisarantis et al., 2019) in which psychological autonomy is strongly associated with well-being. Similarly, research by Inguglia et al. (2014) confirms that for emerging adults, autonomy and relatedness are fundamental needs linked to their health and psychological well-being. Meanwhile, González et al. (2015) showed that perceived support for autonomy in athletic activities was a positive predictor of subjective vitality and positive affect in emerging adults, as well as being a predictor of satisfaction for three indicators of psychological well-being: competence, autonomy, and relatedness.

Likewise, for all three pathways, we find a significant correlation between the *purpose in life* dimension on the well-being scale and all dimensions on the autonomy scale. This observation is compatible with the opinion, already expressed by numerous researchers, that autonomy is of great relevance to the life choices made by emerging adults regarding education and employment. Their decision-making, which defines their purpose in life, stems from the processes of social self-construction and personal identity consolidation (Campbell and Ungar, 2004; Savickas et al., 2009; Bernaud, 2014; Maree and Twigge, 2016). The result is also coherent with studies reporting an association between young people’s higher levels of well-being and satisfaction with life and their ability to develop purpose in life, attain self-acceptance, and learn to navigate their environments (Cardona et al., 2014). A closer look at socially disadvantaged youth reveals a pathway marked by dependence on institutions, difficulty in the workplace, and poor academic results; all of these tendencies are associated with low levels of psychological well-being (Bernal Romero, 2016; Dixon, 2016; Goyette, 2019).

Returning to the dimension analysis, we determined that the *autonomy* dimension on the well-being scale showed a significant and positive correlation, for all three pathways, with the *context analysis* dimension on the autonomy scale. This result suggests that a greater capacity for context analysis is accompanied by greater autonomy, and vice versa. The ability to analyze a situation, according to Bernal Romero et al. (2020b), indicates the capacity to search for information, mull over proposals, and adopt positions or make decisions in personal, social, and political matters. Similarly, the ability to analyze context, according to Ryff and Singer (2008), is useful for avoiding looking to others for approval in different social settings; these researchers link autonomy to independence and self-determination. In their studies of emerging adults who attend universities, García-Alandete et al. (2018) reinforce this idea by framing the construct of autonomy as self-reliance, separated from the intersubjective experience. However, according to Meléndez et al. (2018), young people will still experience difficulty preserving their individuality in many social situations given that they feel more need for outside approval while their identities are still forming.

According to the above definitions, an emerging adult able to maintain his or her autonomy and individuality in different settings will first have to be able to comprehend, analyze, and adapt to each setting (family, academic, social, other). This construct of autonomy is therefore understood from both intrasubjective and intersubjective viewpoints, as other researchers have proposed (Arnett, 2006; Bekker and Van Assen, 2006; Inguglia et al., 2014; Bernal Romero et al., 2016; Duineveld et al., 2017; Kiang and Bhattacharjee, 2018; Ryff, 2018; Van Petegem et al., 2015).

### Score Comparisons Across Pathways (Specific Objective 2)

Recall that our second specific objective is *to detect any significant differences between emerging adults on different pathways using scores from each dimension on the autonomy questionnaire, and from each dimension on the well-being questionnaire*. Significant differences among emerging adults on different pathways could be identified using both the autonomy and the well-being questionnaires.

A comparison of both total scores and dimension scores on the well-being scale revealed significant differences between the pathways; the highest levels of psychological well-being belonged to the education group, followed by the employment group. The lowest levels of psychological well-being were found in the social disadvantage group. Below, we will examine the most salient differences for each pathway.

Young people in the education group had higher total scores on both the autonomy and well-being scales compared to participants in the other two groups. Similar results have been found in other studies of young university students. Barrantes-Brais and Ureña-Bonilla (2015) and García-Alandete et al. (2018) attribute stronger showings for personal growth and purpose in life to university students; they highlight a positive correlation between purpose in life and overall psychological well-being. Our study has uncovered significant differences between the education group and the other two groups for the *critical thinking* dimension, with the education group showing the highest scores. Well-developed critical thinking skills are associated with greater respect for the self and for one’s opinions and ideas and the ability to express those ideas during a confrontation or a situation requiring an assertive response (Bernal Romero et al., 2020b). As reported by Zepke (2015) and Arnett and Tanner (2016) higher education research has often found high levels of engagement and critical consciousness in students. While both factors might present only in the classroom, these authors believe they can also be studied in broader contexts, such as sociocultural ecosystems in which students establish the links between themselves, their immediate setting, and the wider community.

The employment group’s scores on the autonomy scale highlight a more developed capacity for self-organization than that found in the other pathways, and the difference between the employment and education groups is statistically significant. As stated by various researchers working from a self-construction theory perspective, emerging adults must self-organize in order to maintain stability and continuity in their professional careers; as such, issues in professional development play a fundamental role in how they engineer their lives (Guichard et al., 2012). According to Maree and Twigge (2016), these issues include the importance of social consciousness and promoting social justice in the workplace. In fact, researchers studying young adults have found positive correlations between workplace autonomy and job satisfaction on the one hand, and organizational/workplace commitment on the other (Ng and Feldman, 2010), and these correlations are stronger than in older workers (Zacher and Schmitt, 2016). Nevertheless, self-organization and its role in the education and training of emerging adults that follow the education pathway before becoming employed do not typically concern employers (Pimentel et al., 2016).

Another relevant finding is that the social disadvantage group showed significantly higher levels of socio-political engagement (autonomy scale) compared to both the education and employment groups. Socio-political engagement refers to a person’s ability to make decisions according to that person’s degree of social responsibility and participation in contemporary society (Bernal Romero et al., 2020b). This finding resembles a description by Munson et al. (2013) of young peoples’ interest in getting involved in their communities, helping others, and making sure that other young people in similar situations will receive the attention they needed themselves.

Total scores on the well-being scale are significantly lower for the social difficulty pathway. The lower levels of psychological well-being observed among these participants may be linked to their sense of lack of control over their lives and a more external locus of control, among other factors (Król et al., 2019). Others have observed that attachment strength in relationships is a predictor of well-being in adults (Borelli et al., 2018). This factor may help explain why socially disadvantaged youths, those whose family relationships may not have contributed to the formation of secure attachments, score the lowest on the well-being scale. Multiple authors have reported this tendency among socially disadvantaged youth, whose pathway diverges from those of other emerging adults (Fowler et al., 2011; Munson et al., 2013). Very often, these individuals require additional emotional and social support (Goodkind et al., 2011; Schofield et al., 2016). In many cases, the situation becomes even more critical when emerging adults reach the age of 18 and cannot access their usual social services; at this point, they find themselves at a stark disadvantage compared to young people on other pathways (Arnett, 2000, 2006; Osgood et al., 2010; Munson et al., 2013).

#### Limitations and Future Directions

One of the methodological limitations of this study, and a reason for interpreting results with caution, is that the sample sizes differ substantially among the pathways. For that reason, we replicated the tests (Kruskal–Wallis and one-way ANOVA) with equal sample sizes to ensure data reliability and determined the resulting differences to be the same as those identified in the original groups. Although the demographic variables “country of origin,” “age,” and “sex” were similar for all three groups (see Table 1), we also tested to rule out any potential effects that might skew the results. Our testing, which examined both the well-being and the autonomy scale results, revealed no significant demographic differences between pathways that would have to be controlled for.

Regarding magnitude of differences, effect sizes in this study, unsurprisingly, are small to moderate; Rosnow and Rosenthal (2009) determined that the typical effect sizes in the social sciences are often very small. Additionally, according to Ferguson (2009), these effect sizes may not be indicative of the “true” effect when samples are too large, or when they are not selected randomly.

Another limitation of this study is that the sample was not randomly selected, which is an obstacle to external validation and makes it difficult to extrapolate results to the general population.

Since the sample characteristics have been identified as a limitation, a future study would aim to use a probability sampling method that would guarantee equivalent group sizes while controlling for confounders; in this way, the study could also be expanded to include other countries of interest.

To summarize, this study’s results and their interpretation contribute to our understanding of the different pathways followed by emerging adults and also provide a reference dataset for future studies on autonomy and well-being in young people. We should highlight that the study furnishes data that may be of great interest to professionals aiming to design and target interventions for emerging adults on different pathways.

In fact, some studies indicate that academic success can follow the implementation of strategies promoting development of youth autonomy and self-sufficiency (De Carvalho and De Almeida, 2011; Longas and Riera, 2016). Additionally, psychoeducational support and personalized study programs improve peer and teacher–student relationships, while also fostering an increased sense of responsibility and preparedness for independent living (Antunes and Correia, 2016). Researchers have highlighted the importance of training emerging adults who work or are on the employment pathway in how to develop strategies that promote well-being (Merino et al., 2019). Meanwhile, the autonomy and well-being of socially disadvantaged or vulnerable emerging adults must be nurtured through support for their personal, social, and functional skills and by improving social and academic intervention processes (Antunes and Correia, 2016; Zamith-Cruz et al., 2016; Melendro et al., 2017). Above all else, these individuals require social interventions throughout the emerging adulthood stage; without additional support, many will not attain levels of autonomy and well-being similar to those of young people on other pathways (Munson et al., 2013).

Each of these lines of action will require meticulous and rigorous data acquisition, and additional studies on the development of autonomy and well-being, if we are to successfully tackle the distinct realities faced by young people in our time.

## Data Availability Statement

The datasets generated for this study are available on request to the corresponding author.

## Ethics Statement

The studies involving human participants were reviewed and approved by the Ethics Committee of the Universidad Santo Tomás (Bogotá, Colombia) and the Ethics Committee of the Universidad Nacional de Educación a Distancia (Madrid, Spain). All participants were given a full description of the study and informed that participation was voluntary. Informed consent was obtained. In the case of minors, in addition to their consent, consent from parents or guardians was also obtained.

## Author Contributions

MM coordinated the project and received financial support for the study. DA set up the database and completed the statistical analysis. MM and DA drafted the initial version of the article, which was then revised by all authors. GC and AR-B prepared the introduction and theoretical framework and wrote the discussion section with MM. GC and AR-B reviewed the references section. All authors contributed to the article and approved the submitted version.

## Conflict of Interest

The authors declare that the research was conducted in the absence of any commercial or financial relationships that could be construed as a potential conflict of interest.

## References

[B1] Adler A. Seligman M. E. P. (2016). Using wellbeing for public politic: theory, measurement, and recommendations. *Int. J. Wellbeing* 6 1–35. 10.5502/Ijw.V6i1.429 32285354

[B2] Alivernini F. Cavicchiolo E. Manganelli S. (2019). Support for autonomy at school predicts immigrant adolescents’. Psychological Well-being. *Immigr. Minor. Health* 21 761–766. 10.1007/s10903-018-0839-x 30448930

[B3] Allport G. W. (1961). *Pattern and Growth in Personality.* New York, NY: Reinhart & Winston.

[B4] Antunes M. D. C. P. Correia L. F. L. (2016). “Educar para a autonomia de vida: uma intervenção com crianças/jovens institucionalizados,” in *Fronteiras, diálogos e transições na educação. XIII Congresso da Sociedade Portuguesa de Ciências da Educação (SPCE)*, eds Acevedo C. Presidencia Viseu

[B5] Arnett J. J. (2000). Emerging adulthood: a theory of development from the late teens through the twenties. *Am. Psychol.* 55 469–480. 10.1037/0003-066X.55.5.469 10842426

[B6] Arnett J. J. (2006). “Understanding the new way of coming of age,” in *Emerging adults in America: Coming of age in the 21st century*, eds Arnett J. J. Tanner J. L. (Washington, DC: American Psychological Association), 193–217.

[B7] Arnett J. J. (2007). Emerging adulthood: what is it, and what is it good for? *Child Dev. Perspect.* 1 68–73. 10.1111/j.1750-8606.2007.00016.x

[B8] Arnett J. J. (2015). *Emerging Adulthood: The Winding road From the Late Teens Through the Twenties*, 2nd Edn New York, NY: Oxford University Press.

[B9] Arnett J. J. Tanner J. L. (2016). “The emergence of emerging adulthood: the new life stage between adolescence and young adulthood,” in *Handbook of Youth and Young Adulthood*, ed. Furlong A. (London: Routledge), 50–56.

[B10] Barrantes-Brais K. Ureña-Bonilla P. (2015). Bienestar psicológico y bienestar subjetivo en estudiantes universitarios costarricenses. *Rev. Int. Psicol. Educ.* 17 101–123.

[B11] Bekker M. H. J. Van Assen M. A. L. M. (2006). A short form of the autonomy scale: properties of the autonomy-connectedness scale. *J. Pers. Assess* 86 51–60. 10.1207/s15327752jpa8601_07 16436020

[B12] Berger K. S. (2016). *Psicología del Desarrollo. Infancia & Adolescencia.* Madrid: Editorial Médica Panamericana.

[B13] Bernal Romero T. (2016). *El tránsito a la vida Adulta de Jóvenes Egresados del Sistema de Protección en Colombia: Trayectorias, Fuentes de Resiliencia e Intervenciones Socioeducativas.* dissertation’s thesis, UNED, Madrid.

[B14] Bernal Romero T. Daza Pinzón C. Jaramillo Acosta P. (2016). Satisfacción con la vida y resiliencia en jóvenes en extraedad escolar. *Revista Iberoamericana De Psicología*. 8 43–53.

[B15] Bernal Romero T. Melendro M. Charry C. (2020a). Transition to adulthood autonomy scale for young people: design and validation. *Front. Psychol.* 11:457. 10.3389/fpsyg.2020.00457 32265784 PMC7100080

[B16] Bernal Romero T. Melendro M. Charry C. L. Goig R. M. (2020b). *La influencia de la familia y la educación en la autonomía de los jóvenes: una revisión sistemática*. *Bordón Revista de pedagogía*. 72 29–44. 10.13042/Bordon.2020.76175

[B17] Bernaud J. L. (2014). “Career counseling and life meaning: a new perspective of life designing for research and applications,” in *The Construction of the Identity in 21st Century: A Festschrift for Jean Guichard*, eds Di Fabio A. Bernaud J. L. (New York, NY: Nova Science), 29–40.

[B18] Borelli J. L. Brugnera A. Zarbo C. Rabboni M. Bondi E. Tasca G. A. (2018). Attachment comes of age: adolescents’ narrative coherence and reflective functioning predict well-being in emerging adulthood. *Attach. Hum. Dev.* 21 332–351. 10.1080/14616734.2018.1479870 29865892

[B19] Cáliz N. Jaimes M. Martínez L. Fandino V. (2013). Autonomía y calidad de vida de adolescentes en condición de desplazamiento forzoso en la localidad de Suba. Bogotá, D. C. *Avances Enfermería* 31 87–102. 10.15446/av.enferm

[B20] Cameron C. Hollingworth K. Schoon I. van Santen E. Schröer W. Ristikari T. (2018). Care leavers in early adulthood: how do they fare in Britain, Finland and Germany? *Child. Youth Serv. Rev.* 87 163–172. 10.1016/j.childyouth.2018.02.031

[B21] Campbell C. Ungar M. (2004). Constructing a life that works, part 2: an approach to practice. *Career Dev. Q.* 53 28–40. 10.1002/j.2161-0045.2004.tb00653.x

[B22] Campione-Barr N. Lindell A. K. Short S. D. Greer K. B. Drotar S. D. (2015). First- and second-born adolescents’ decision-making autonomy throughout adolescence. *J. Adolesc.* 45 250–262. 10.1016/j.adolescence.2015.10.009 26519875

[B23] Campos G. Goig R. Cuenca M. E. (2020). Relevance of the social support network for the emancipation of young adults leaving residential care. *Electron. J. Res. Educ. Psychol.* 18 27–54. 10.25115/ejrep.v18i50.2599

[B24] Cardona D. Rodríguez J. Osorio Tamayo L. Moreno Carmona N. D. (2014). Construcción del bienestar juvenil en las actuales dinámicas de socialización. *Rev. Colomb. Cien. Soc.* 5 77–98.

[B25] Chatzisarantis N. L. D. Ada E. N. Ahmadi M. Caltabiano N. Wang D. Thogersen-Ntoumani C. (2019). Differential effects of perceptions of equal, favourable and unfavourable autonomy support on educational and well-being outcomes. *Contemp. Educ. Psychol.* 58 33–43. 10.1016/j.cedpsych.2019.02.002

[B26] Cohen J. (1992). A power primer. *Psychol. Bull.* 112 155–159. 10.1037/0033-2909.112.1.155 19565683

[B27] Cuenca E. Campos G. Goig R. M. (2018). Young people’s transition from residential care to adulthood; the family’s role. *Educación* 21 321–344. 10.5944/educXX1.16510

[B28] Davies J. Kelly D. Hannigan B. (2015). Autonomy and dependence: a discussion paper on decision-making in teenagers and young adults undergoing cancer treatment. *J. Adv. Nurs.* 71 2031–2040. 10.1111/jan.12669 25884430

[B29] De Carvalho J. C. B. De Almeida S. F. C. (2011). Desenvolvimento moral no ensino médio: concepções de professores e autonomia dos alunos. *Psicol. Argum.* 29 187–199.

[B30] Deci E. L. Ryan R. M. (2008). Hedonia, eudaimonia, and well-being: an introduction. *J. Happiness Stud.* 9 1–11. 10.1007/S10902-006-9018-1

[B31] Delbosc A. Vella-Brodrick D. (2015). The role of transport in supporting the autonomy of young adults. *Trans. Res. Part F Traffic Psychol. Behav.* 33 97–105. 10.1016/j.trf.2015.03.011

[B32] Di Fabio A. Kenny M. E. (2016). Promoting well-being: the contribution of emotional intelligence. *Front. Psychol.* 7:1182. 10.3389/fpsyg.2016.01182 27582713 PMC4987330

[B33] Díaz D. Rodríguez-Carvajal R. Blanco A. Moreno-Jiménez B. Gallardo I. Valle C. (2006). Adaptación española de las escalas de bienestar psicológico de Ryff. *Psicothema* 18 572–577. 17296089

[B34] Dixon J. (2016). Opportunities and challenges: supporting journeys into education and employment for young people leaving care in England. *Rev. Español. Pedag.* 263 13–29.

[B35] Duineveld J. Parker P. Ryan R. Ciarrochi J. Salmela-Aro K. (2017). The link between perceived maternal and paternal autonomy support and adolescent well-being across three major educational transitions. *Dev. Psychol.* 53 1978–1994. 10.1037/dev0000364 28805437

[B36] European Policy Centre (2014). Challenges and New Beginnings: Priorities for the EU’s new Leadership. Challenge Europe- Challenges and new beginnings: Priorities for the EU’s new leadership. Avaliable at: http://aei.pitt.edu/56408/

[B37] Ferguson C. J. (2009). An effect size primer: a guide for clinicians and researchers. *Profess. Psychol.* 40 532–538. 10.1037/a0015808

[B38] Fotiadis A. Abdulrahman K. Spyridou A. (2019). The mediating roles of psychological autonomy, competence and relatedness on work-life balance and well-being. *Front. Psychol.* 10:1267. 10.3389/fpsyg.2019.01267 31191420 PMC6549400

[B39] Fowler P. J. Toro P. A. Miles B. W. (2011). Emerging adulthood and leaving foster care: settings associated with mental health. *Am. J. Community Psychol.* 47 335–348. 10.1007/s10464-010-9401-2 21184169

[B40] Garberoglio C. L. Schoffstall S. Cawthon S. Bond M. Caemmerer J. M. (2017). The antecedents and outcomes of autonomous behaviors: modeling the role of autonomy in achieving sustainable employment for deaf young adults. *J. Dev. Phys. Disabil.* 29 107–129. 10.1007/s10882-016-9492-2

[B41] García-Alandete J. Rubio-Belmonte C. Soucase B. A. (2018). A replication of the reker and cousins’ study about the complementarity between the purpose-in-life test (PIL) and the seeking of noetic goals (SONG) among spanish young people. *J. Happiness Stud.* 19 81–97. 10.1007/s10902-016-9810-5

[B42] García-Castilla F. J. Melendro M. Blaya C. (2018). Preferencias, renuncias y oportunidades en la práctica de ocio de los jóvenes vulnerables. *Pedagogía Social. Revista Interuniversitaria* 31 21–32. 10.7179/PSRI_2018.31.02

[B43] González L. Castillo I. García-Merita M. Balaguer I. (2015). Autonomy support, psychological needs satisfaction and well-being: invariance of a structural model in soccer players and dancers. *Rev. Psicol. Dep.* 24 121–129.

[B44] Goodkind S. Schelbe L. A. Shook J. J. (2011). Why youth leave care: understandings of adulthood and transition success and challenges among youth aging out of child welfare. *Child. Youth Serv. Rev.* 33 1039–1048. 10.1016/j.childyouth.2011.01.010

[B45] Goyette M. (2019). “Leaving care and the transition to adulthood,” in *Leaving Care and the Transition to Adulthood: International Contributions to Theory, Research, and Practice*, eds Mann-Feder V. R. Goyette M. (Oxford, UK: Oxford University Press), 329–345.

[B46] Guichard J. Pouyaud J. De Calan C. Dumora B. (2012). Identity construction and career development interventions with emerging adults. *J. Vocat. Behav.* 81 52–55. 10.1016/j.jvb.2012.04.004

[B47] Hausler M. Strecker C. Huber A. Brenner M. Höge T. Höfer S. (2017). Distinguishing relational aspects of character strengths with subjective and psychological well-being. *Front. Psychol.* 8:1159. 10.3389/fpsyg.2017.01159 28744245 PMC5504157

[B48] Inguglia C. Ingoglia S. Liga F. Lo Coco A. Lo Cricchio M. (2014). Autonomy and relatedness in adolescence and emerging adulthood: relationships with parental support and psychological distress. *J. Adult Dev.* 22 1–13. 10.1007/s10804-014-9196-8

[B49] INJUVE (2017). *Informe de Juventud en España.* Madrid: INJUVE.

[B50] Jorgensen N. Nelson L. (2018). Moving toward and away from others: social orientations in emerging adulthood. *J. Appl. Dev. Psychol.* 58 66–76. 10.1016/j.appdev.2018.09.001

[B51] Joshanloo M. (2019). Investigating the relationships between subjective well-being and psychological well-being over two decades. *Emotion* 19 183–187. 10.1037/emo0000414 29494203

[B52] Kiang L. Bhattacharjee K. (2018). Developmental change and correlates of autonomy in Asian American Adolescents. *Jo. Youth Adolesc.* 48 410–421. 10.1007/s10964-018-0909-3 30088130

[B53] Król M. E. Kliś A. Kustra M. Szymañski K. (2019). Is knowledge of family history related to psychosocial functioning? Comparison of adolescents living in institutional care and adolescents living with their biological families. *Eur J. Dev. Psychol.* 16 464–475. 10.1080/17405629.2018.1447460

[B54] Legault L. Ray K. Hudgins A. Pelosi M. Shannon W. (2017). Assisted versus asserted autonomy satisfaction: their unique associations with wellbeing, integration of experience, and conflict negotiation. *Motivat. Emot.* 41 1–21. 10.1007/s11031-016-9593-3

[B55] Liga F. Lo Coco A. Musso P. Inguglia C. Costa S. Lo Cricchio M. G. (2018). Parental psychological control, autonomy support and Italian emerging adult’s psychosocial well-being: a cluster analytic approach. *Eur. J. Dev. Psychol.* 17 37–55. 10.1080/17405629.2018.1532887

[B56] Longas J. Riera J. (2016). Resultados del observatorio transición escuela-trabajo y monitoreo de la red socioeducativa de Sant Vicenç dels Horts para el éxito escolar y el empoderamiento de los jóvenes. *Bordón* 68 103–120. 10.13042/Bordon.2016.48837

[B57] López M. Santos I. Bravo A. del Valle J. F. (2013). El proceso de transición a la vida adulta de jóvenes acogidos en el sistema de protección infantil. *Anal. Psicol.* 29 187–196. 10.6018/analesps.29.1.130542

[B58] Malinauskas R. Dumciene A. (2017). Psychological wellbeing and self-esteem in students across the transition between secondary school and university: a longitudinal study. *Psihologija* 50 21–36. 10.2298/PSI160506003M

[B59] Maree J. G. Twigge A. (2016). Career and self-construction of emerging adults: the value of life designing. *Front. Psychol.* 6:2041. 10.3389/fpsyg.2015.02041 26793152 PMC4707554

[B60] Mateo J. (2004). “La investigación ex post-facto,” in *Metodología de la Investigación Educativa*, ed. Bisquerra R. (Madrid: La Muralla), 195–230.

[B61] Mayordomo T. Sales A. Satorres E. Meléndez J. C. (2016). Bienestar psicológico en función de la etapa de vida, el sexo y su interacción. *Pensam. Psicol.* 14 101–112. 10.11144/Javerianacali.PPSI14-2.bpfe

[B62] Meléndez J. C. Agustí A. I. Delhom I. Reyes M. F. Satorres E. (2018). Bienestar subjetivo y psicológico: comparación de jóvenes y adultos mayores. *Summa Psicol. UST* 15 18–24. 10.18774/0719-448x.2018.15.335

[B63] Melendro M. De-Juanas Oliva A. Rodriguez-Bravo A. E. (2017). Deficiencies in socio-educational intervention with families of adolescents in risk of exclusion. *Bordón. Revista de Pedagogía* 69 123–138. 10.13042/Bordon.2016.48596

[B64] Merino M. D. Privado J. Arnaiz R. (2019). Is There Any Relationship between unemployment in Young Graduates and Psychological Resources? An empirical research from the conservation of resources theory. *J. Work Organ. Psychol.* 35 1–8. 10.5093/jwop2019a1

[B65] Muñoz-López S. Alvarado S. (2011). Autonomía en movimiento: reflexión desde las prácticas políticas alternativas de jóvenes en Colombia. *Revista Latinoamericana de Ciencias Sociales, Niñez y Juventud* 1 115–128.

[B66] Munson M. R. Lee B. R. Miller D. Cole A. Nedelcu C. (2013). Emerging adulthood among former system youth: the ideal versus the real. *Children Youth Serv. Rev.* 35 923–929. 10.1016/j.childyouth.2013.03.003

[B67] Ng T. W. Feldman D. C. (2010). The relationships of age with job attitudes: a meta-analysis. *Pers. Psychol.* 63 677–718. 10.1111/j.1744-6570.2010.01184.x

[B68] Osgood D. W. Foster M. Courtney M. E. (2010). Vulnerable populations and the transition to adulthood. *Future Child* 20 209–229. 10.1353/foc.0.0047 20364628

[B69] Oudekerk B. Allen J. Hessel E. Molloy L. (2015). The cascading development of autonomy and relatedness from adolescence to adulthood. *Child Dev.* 86 472–485. 10.1111/cdev.12313 25345623 PMC4376599

[B70] Pardo A. San Martin R. (2010). *Análisis de Datos en Ciencias Sociales y de la Salud II.* Madrid: Síntesis.

[B71] Parron A. (2014). Autonomy issues for young adults dealing with psychic disorders. *ALTER Eur. J. Disabil. Res. Rev. Eur. Rech. Hand.* 8 245–255. 10.1016/j.alter.2014.09.001

[B72] Pimentel C. Silva H. Ferreira Dias M. Amorim M. (2016). “Transversal entrepreneurial competencies for youth employability: a GAP analysis,” in *Proceedings of Business Sustainability*, Póvoa de Varzim, 2100.

[B73] Posada Á (2013). Autoridad y autonomía en la crianza. *Precop Sociedad Colombiana de Pediatría.* 4 5–15.

[B74] Reis H. T. Sheldon K. M. Gable S. L. Roscoe J. Ryan R. M. (2000). Daily well-being: the role of autonomy, competence, and relatedness. *Persona. Soc. Psychol. Bull.* 26 419–435. 10.1177/0146167200266002

[B75] Rifkin J. (2011). *The Third Industrial Revolution: How Lateral Power Is Transforming Energy, the Economy, and the World.* New York, NY: Palgrave Macmillan.

[B76] Rosnow R. L. Rosenthal R. (2009). Effect sizes for experimenting psychologists: correction to rosnow and rosenthal (2003). *Can. J. Exp. Psychol.* 63 123–123. 10.1037/a0015528 14596479

[B77] Ryff C. D. (1989). Happiness is everything, or is it? Explorations on the meaning of psychological well-being. *J. Personal. Soc. Psychol.* 57 1069–1081. 10.1037/0022-3514.57.6.1069

[B78] Ryff C. D. (1991). Possible selves in adulthood and old age: a tale of shifting horizons. *Psychol. Aging* 6 286–295. 10.1037/0882-7974.6.2.286 1863398

[B79] Ryff C. D. (2018). Entrepreneurship and eudaimonic well-being: five venues for new science. *J. Bus. Ventu.* 34 646–663. 10.1016/j.jbusvent.2018.09.003 31105380 PMC6516495

[B80] Ryff C. D. Keyes C. L. M. (1995). Structure of psychological well-being revisited. *J. Personal. Soc. Psycol.* 69 719–727. 10.1037/0022-3514.69.4.719 7473027

[B81] Ryff C. D. Singer B. H. (2008). Know thyself and become what you are: a eudaimonic approach to psychological well-Being. *J. Happiness Stud.* 9 13–39. 10.1007/S10902-006-9019-0

[B82] Savickas M. L. Nota L. Rossier J. Dauwalder J. P. Duarte M. E. Guichard J. (2009). Life designing: a paradigm for career construction in the 21st century. *J. Vocat. Behav.* 75 239–250. 10.1016/j.jvb.2009.04.004

[B83] Schoon I. Lyons-Amos M. (2016). Diverse pathways in becoming an adult: the role of structure, agency and context. *Res. Soc. Stratif. Mobil.* 46A 11–20. 10.1016/j.rssm.2016.02.008

[B84] Schofield G. Larsson B. Ward E. (2016). Risk, resilience and identity construction in the life narratives of young people leaving residential care. *Child Fam. Soc. Work* 22 782–791. 10.1111/cfs.12295

[B85] Schüler J. Sheldon K. M. Prentice M. Halusic M. (2014). Do some people need autonomy more than others? Implicit dispositions toward autonomy moderate the effects of felt autonomy on well-being. *J. Pers.* 84 5–20. 10.1111/jopy.12133 25223431

[B86] Settersten R. A. Ottusch T. M. Schneider B. (2015). “Becoming adult: meanings of markers to adulthood,” in *Emerging Trends in the Social and Behavioral Sciences*, eds Scott R. Kosslyn S. (Hoboken, NJ: John Wiley & Sons, Inc), 1–16. 10.1002/9781118900772.etrds0021

[B87] Singer E. R. Berzin S. C. (2014). Early adult identification among youth with foster care experience: implications for emerging adulthood. *J. Public Child Welfare* 9 65–87. 10.1080/15548732.2014.983290

[B88] Soldevila A. Peregrino A. Oriol X. Filella G. (2013). Evaluation of residential care from the perspective of older adolescents in care. The need for a new construct: optimum professional proximity. *Child Fam. Soc. Work* 18 285–293. 10.1111/j.1365-2206.2012.00843.x

[B89] Van Petegem S. Vansteenkiste M. Soenens B. Beyers W. Aelterman N. (2015). Examining the longitudinal association between oppositional defiance and autonomy in adolescence. *Dev. Psychol.* 51 67–74. 10.1037/a0038374 25419798

[B90] Waterman A. S. Schwartz S. J. Zamboanga B. L. Ravert R. D. Williams M. K. Bede Agocha V. (2010). The questionnaire for eudaimonic well-being: psychometric properties, demographic comparisons, and evidence of validity. *J. Posit. Psychol.* 5 41–61. 10.1080/17439760903435208 PMC8317967 34326891

[B91] Wood D. Crapnell T. Lau L. Bennett A. Lotstein D. Ferris M. (2018). “Emerging adulthood as a critical stage in the life course,” in *Handbook of Life Course Health Development*, eds Halfon N. Forrest C. Lerner R. Faustman E. (Cham: Springer), 123–143. 10.1007/978-3-319-47143-3_7 31314293

[B92] Zacher H. Schmitt A. (2016). Work characteristics and occupational well-being: the role of age. *Front. Psychol.* 7:1411. 10.3389/fpsyg.2016.01411 27713711 PMC5031689

[B93] Zamith-Cruz J. Z. Lopes A. Carvalho M. D. L. D. D. (2016). Educação para a autonomia em instituições de crianças e jovens: o que nos dizem as narrativas dos profissionais. *Pesqu. Qual.* 4 353–367.

[B94] Zepke N. (2015). Student engagement research: thinking beyond the mainstream. *High. Educ. Res. Dev.* 34 1311–1323. 10.1080/07294360.2015.1024635

